# Ethyl 3-(4-methyl­benzene­sulfonamido)thieno[2,3-*b*]pyridine-2-carboxyl­ate

**DOI:** 10.1107/S1600536809000269

**Published:** 2009-01-08

**Authors:** Wen-qin Zhang, Ren-lin Zheng, Hang Song, Sheng-Yong Yang, Luo-Ting Yu

**Affiliations:** a State Key Laboratory of Biotherapy and Cancer Center, West China Hospital, West China Medical School, Sichuan University, Chengdu 610041, People’s Republic of China; b Department of Pharmaceutical and Biological Engineering, School of Chemical Engineering, Sichuan University, Chengdu 610065, People’s Republic of China

## Abstract

The thieno[2,3-*b*]pyridine ring system of the title compound, C_17_H_16_N_2_O_4_S_2_, is essentially planar, the amino and carbonyl groups being nearly coplanar with the heterocyclic ring system. There are two N—H⋯O hydrogen-bonding inter­actions involving the same N—H donor set and two different acceptors, one in an intra­molecular bond helping to fix the mol­ecular geometry and the other defining a dimeric structure around the symmetry centre at (0, 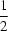, 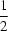).

## Related literature

For general background, see: Litvinov *et al.* (2005 ▶). 
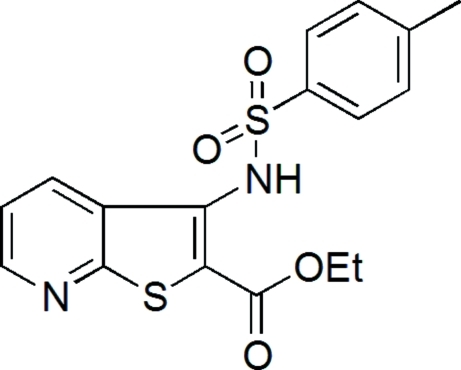


## Experimental

### 

#### Crystal data

C_17_H_16_N_2_O_4_S_2_
*M* *_r_* = 376.46
Orthorhombic, 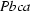

*a* = 14.809 (4) Å
*b* = 11.892 (3) Å
*c* = 19.494 (5) Å
*V* = 3433.1 (15) Å^3^
*Z* = 8
Mo *K*α radiation
μ = 0.34 mm^−1^
*T* = 293 (2) K
0.46 × 0.44 × 0.42 mm

#### Data collection

Enraf–Nonius CAD-4 diffractometer
Absorption correction: spherical [modified Dwiggins (1975 ▶) interpolation procedure] *T*_min_ = 0.861, *T*_max_ = 0.872
3590 measured reflections
3075 independent reflections
2032 reflections with *I* > 2σ(*I*)
*R*_int_ = 0.005
3 standard reflections every 200 reflections intensity decay: 3.0%

#### Refinement

*R*[*F* ^2^ > 2σ(*F* ^2^)] = 0.055
*wR*(*F* ^2^) = 0.186
*S* = 1.13
3075 reflections
233 parameters
1 restraint
H atoms treated by a mixture of independent and constrained refinement
Δρ_max_ = 0.36 e Å^−3^
Δρ_min_ = −0.74 e Å^−3^

### 

Data collection: *DIFRAC* (Gabe & White, 1993 ▶); cell refinement: *DIFRAC*; data reduction: *NRCVAX* (Gabe *et al.*, 1989 ▶); program(s) used to solve structure: *SHELXS97* (Sheldrick, 2008 ▶); program(s) used to refine structure: *SHELXL97* (Sheldrick, 2008 ▶); molecular graphics: *ORTEP-3* (Farrugia, 1997 ▶); software used to prepare material for publication: *SHELXL97*.

## Supplementary Material

Crystal structure: contains datablocks global, I. DOI: 10.1107/S1600536809000269/bg2232sup1.cif

Structure factors: contains datablocks I. DOI: 10.1107/S1600536809000269/bg2232Isup2.hkl

Additional supplementary materials:  crystallographic information; 3D view; checkCIF report

## Figures and Tables

**Table 1:** Hydrogen-bond geometry (Å, °)

*D*—H⋯*A*	*D*—H	H⋯*A*	*D*⋯*A*	*D*—H⋯*A*
N1—H1*N*⋯O4	0.95 (3)	2.22 (3)	2.867 (4)	125 (3)
N1—H1*N*⋯O2^i^	0.95 (3)	2.21 (3)	3.033 (4)	145 (3)

Symmetry code: (i) 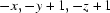.

**Fig. 1.**
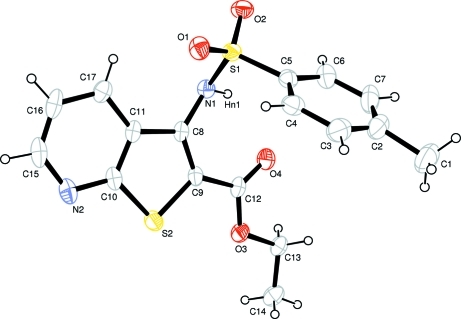
The molecular structure of the title compound, with displacement ellipsoids drawn at the 30% probability level.

**Fig. 2.**
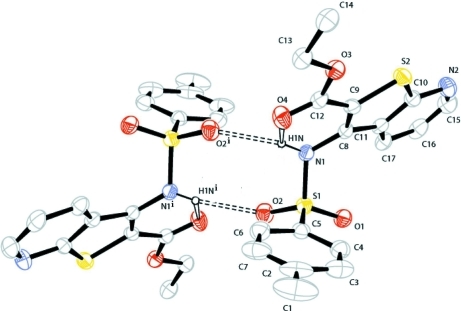
View of the dimeric structure in the title compound. Hydrogen bonds are shown as hollow bonds (intermolecular ones as dashed lines, intramolecular ones in full lining). Symmetry code: (i) -x, -y+1, -z+1.

## supplementary crystallographic information

### Comment

Thieno[2,3-*b*]pyridine derivatives are of great importance owing to their wide biological properties (Litvinov *et al.*,2005). The title compound is one of the key intermediates in our synthetic investigations of antitumor drugs. We report here its crystal structure.

The thieno[2,3-*b*]pyridine ring system of the title compound C_17_H_16_N_2_O_4_S_2_, (Fig.1) is essentially planar, with the amino and the carbonyl groups being nearly coplanar with the heterocyclic ring system.

There are two main N-H···O H-bonding interactions (Table 1), involving the same N1-H1N donor set and two different acceptors, O4 (in an intramolecular bond fixing the molecular geometry) and O2 ^i^, i: -x, -y+1, -z+1, defining a dimeric structure around the symmetry centre at (0,0.5,0.5) (Fig.2)

### Experimental

A solution of ethyl 3-amino-4*H*-thieno[2,3-*b*]pyridine -2-carboxylate(2 g, 9 mmol), *p*-toluenesulfonyl chloride (5.2 g, 27 mmol) and pyridine (3.7 ml, 45 mmol) in dichloromethane (100 ml) was stirred until the reaction was complete. The reaction mixture was washed twice with a saturated aqueous solution of CuSO_4_ and once with water. The organic layer was dried and concentrated *in vacuum* and the resulting residue was purified by crystallization from dichloromethane to yield a white solid (3.1 g, 91%). Crystals suitable for X-ray analysis were obtained by slow evaporation from a solution of dichloromethane and petroleum (1:2).

### Refinement

The crystal used for data collection was ground into a spheroidal shape in order to facilitate the absorption correction. This was made through an interpolation procedure with local modifications to the one reported by Dwiggins, 1975. muR values in the range 0-2.5 were taken from the International Tables for X-ray Crystallography (1992, Vol. C, p. 523), while those for muR in the range 2.6-10.0. were taken from International Tables for X-ray Crystallography (1959, Vol II, p. 302)

H atoms of the amino group were located in a difference map and refined freely. The remaining H atoms were positioned geometrically (C—H = 0.93–0.97 Å) and refined using a riding model, with *U*ĩso~(H) = 1.2–1.5*U*~eq~(C).

### Crystal data

**Table:**

C_17_H_16_N_2_O_4_S_2_	*F*(000) = 1568
*M**_r_* = 376.46	*D*_x_ = 1.457 Mg m^−^^3^
Orthorhombic, *P**b**c**a*	Mo *K*α radiation, λ = 0.71073 Å
Hall symbol: -P 2ac 2ab	Cell parameters from 24 reflections
*a* = 14.809 (4) Å	θ = 4.5–7.7°
*b* = 11.892 (3) Å	µ = 0.34 mm^−^^1^
*c* = 19.494 (5) Å	*T* = 293 K
*V* = 3433.1 (15) Å^3^	Block, colourless
*Z* = 8	0.46 × 0.44 × 0.42 mm

### Data collection

**Table:**

Enraf–Nonius CAD-4 diffractometer	2032 reflections with *I* > 2σ(*I*)
Radiation source: fine-focus sealed tube	*R*_int_ = 0.005
graphite	θ_max_ = 25.6°, θ_min_ = 2.1°
ω/2θ scans	*h* = −17→10
Absorption correction: for a sphere (interpolation; International Tables for X-ray Crystallography, 1959, Vol II, p. 302; 1992, Vol. C, p. 523); Table 5.3.6 B for muR in the range 2.6-10.0. The interpolation procedure of C.W.Dwiggins Jr (Acta Cryst.(1975) A31,146-148) is used with some modification.	*k* = −2→14
*T*_min_ = 0.861, *T*_max_ = 0.872	*l* = −13→23
3590 measured reflections	3 standard reflections every 200 reflections
3075 independent reflections	intensity decay: 3.0%

### Refinement

**Table:**

Refinement on *F*^2^	Secondary atom site location: difference Fourier map
Least-squares matrix: full	Hydrogen site location: mixed
*R*[*F*^2^ > 2σ(*F*^2^)] = 0.055	H atoms treated by a mixture of independent and constrained refinement
*wR*(*F*^2^) = 0.186	*w* = 1/[σ^2^(*F*_o_^2^) + (0.0996*P*)^2^ + 1.4444*P*] where *P* = (*F*_o_^2^ + 2*F*_c_^2^)/3
*S* = 1.13	(Δ/σ)_max_ < 0.001
3075 reflections	Δρ_max_ = 0.36 e Å^−^^3^
233 parameters	Δρ_min_ = −0.74 e Å^−^^3^
1 restraint	Extinction correction: *SHELXL97* (Sheldrick, 2008), Fc^*^=kFc[1+0.001xFc^2^λ^3^/sin(2θ)]^-1/4^
Primary atom site location: structure-invariant direct methods	Extinction coefficient: 0.0175 (17)

### Special details

**Table:**

Geometry. All e.s.d.'s (except the e.s.d. in the dihedral angle between two l.s. planes) are estimated using the full covariance matrix. The cell e.s.d.'s are taken into account individually in the estimation of e.s.d.'s in distances, angles and torsion angles; correlations between e.s.d.'s in cell parameters are only used when they are defined by crystal symmetry. An approximate (isotropic) treatment of cell e.s.d.'s is used for estimating e.s.d.'s involving l.s. planes.
Refinement. Refinement of *F*^2^ against ALL reflections. The weighted *R*-factor *wR* and goodness of fit *S* are based on *F*^2^, conventional *R*-factors *R* are based on *F*, with *F* set to zero for negative *F*^2^. The threshold expression of *F*^2^ > σ(*F*^2^) is used only for calculating *R*-factors(gt) *etc*. and is not relevant to the choice of reflections for refinement. *R*-factors based on *F*^2^ are statistically about twice as large as those based on *F*, and *R*- factors based on ALL data will be even larger.

### Fractional atomic coordinates and isotropic or equivalent isotropic displacement parameters (Å^2^)

**Table:**

	*x*	*y*	*z*	*U*_iso_*/*U*_eq_	
S1	0.09964 (6)	0.61780 (8)	0.44511 (5)	0.0446 (3)	
S2	−0.09888 (7)	0.97224 (9)	0.42142 (5)	0.0519 (3)	
O1	0.14122 (18)	0.6604 (2)	0.38494 (13)	0.0557 (7)	
O2	0.09322 (18)	0.4988 (2)	0.45432 (15)	0.0578 (8)	
O3	−0.12382 (18)	0.9327 (2)	0.56425 (12)	0.0497 (7)	
O4	−0.0664 (2)	0.7593 (3)	0.57213 (13)	0.0617 (8)	
N1	−0.0053 (2)	0.6628 (3)	0.44558 (15)	0.0422 (7)	
H1N	−0.033 (3)	0.642 (3)	0.4875 (12)	0.067 (13)*	
N2	−0.0759 (3)	0.9677 (3)	0.28514 (19)	0.0651 (10)	
C1	0.2655 (4)	0.8311 (6)	0.6960 (4)	0.129 (3)	
H1A	0.2173	0.8723	0.7172	0.194*	
H1B	0.3126	0.8821	0.6828	0.194*	
H1C	0.2890	0.7769	0.7278	0.194*	
C2	0.2296 (3)	0.7703 (6)	0.6323 (3)	0.0842 (18)	
C3	0.2462 (3)	0.8165 (5)	0.5677 (3)	0.0783 (15)	
H3	0.2827	0.8798	0.5635	0.094*	
C4	0.2083 (3)	0.7678 (3)	0.5100 (2)	0.0578 (11)	
H4	0.2197	0.7975	0.4668	0.069*	
C5	0.1529 (2)	0.6741 (3)	0.51725 (19)	0.0469 (9)	
C6	0.1372 (3)	0.6279 (4)	0.5808 (2)	0.0622 (11)	
H6	0.1006	0.5646	0.5850	0.075*	
C7	0.1755 (3)	0.6751 (5)	0.6376 (2)	0.0826 (16)	
H7	0.1652	0.6433	0.6805	0.099*	
C8	−0.0288 (2)	0.7731 (3)	0.42354 (17)	0.0399 (8)	
C9	−0.0649 (2)	0.8527 (3)	0.46538 (18)	0.0417 (8)	
C10	−0.0648 (2)	0.9136 (4)	0.34493 (19)	0.0495 (10)	
C11	−0.0285 (2)	0.8071 (3)	0.35295 (17)	0.0434 (9)	
C12	−0.0836 (2)	0.8412 (4)	0.53894 (18)	0.0446 (9)	
C13	−0.1472 (3)	0.9290 (3)	0.63719 (18)	0.0524 (10)	
H13A	−0.0931	0.9208	0.6648	0.063*	
H13B	−0.1867	0.8657	0.6464	0.063*	
C14	−0.1939 (3)	1.0370 (4)	0.6541 (2)	0.0623 (11)	
H14A	−0.1538	1.0988	0.6456	0.093*	
H14B	−0.2112	1.0367	0.7016	0.093*	
H14C	−0.2468	1.0446	0.6260	0.093*	
C15	−0.0523 (3)	0.9075 (5)	0.2299 (2)	0.0724 (14)	
H15	−0.0606	0.9403	0.1870	0.087*	
C16	−0.0170 (3)	0.8024 (5)	0.2319 (2)	0.0662 (13)	
H16	−0.0015	0.7670	0.1910	0.079*	
C17	−0.0036 (2)	0.7466 (4)	0.29326 (19)	0.0511 (10)	
H17	0.0202	0.6743	0.2952	0.061*	

### Atomic displacement parameters (Å^2^)

**Table:**

	*U*^11^	*U*^22^	*U*^33^	*U*^12^	*U*^13^	*U*^23^
S1	0.0471 (6)	0.0449 (6)	0.0417 (6)	0.0037 (4)	0.0058 (4)	−0.0009 (4)
S2	0.0528 (6)	0.0546 (7)	0.0483 (6)	0.0089 (5)	−0.0010 (4)	0.0069 (5)
O1	0.0528 (15)	0.0707 (19)	0.0435 (16)	0.0033 (14)	0.0104 (12)	0.0073 (13)
O2	0.0690 (19)	0.0428 (17)	0.0617 (19)	0.0040 (13)	0.0060 (13)	−0.0015 (13)
O3	0.0542 (15)	0.0584 (17)	0.0365 (14)	0.0043 (13)	0.0087 (11)	−0.0027 (12)
O4	0.084 (2)	0.066 (2)	0.0350 (15)	0.0072 (16)	0.0038 (14)	0.0072 (14)
N1	0.0420 (16)	0.0428 (17)	0.0418 (17)	−0.0035 (13)	0.0015 (13)	0.0029 (13)
N2	0.072 (2)	0.075 (3)	0.048 (2)	0.0039 (19)	−0.0066 (17)	0.0199 (19)
C1	0.093 (4)	0.175 (7)	0.120 (5)	0.051 (4)	−0.052 (4)	−0.086 (5)
C2	0.047 (3)	0.123 (5)	0.083 (4)	0.031 (3)	−0.024 (2)	−0.047 (3)
C3	0.053 (3)	0.084 (4)	0.098 (4)	0.010 (2)	−0.017 (3)	−0.028 (3)
C4	0.050 (2)	0.049 (2)	0.074 (3)	0.0013 (18)	−0.005 (2)	−0.002 (2)
C5	0.043 (2)	0.050 (2)	0.048 (2)	0.0082 (17)	0.0021 (16)	0.0002 (17)
C6	0.053 (2)	0.079 (3)	0.055 (3)	−0.001 (2)	−0.0040 (19)	0.007 (2)
C7	0.058 (3)	0.140 (5)	0.050 (3)	0.026 (3)	−0.010 (2)	−0.012 (3)
C8	0.0349 (17)	0.053 (2)	0.0318 (18)	−0.0077 (15)	−0.0022 (14)	0.0041 (15)
C9	0.0363 (17)	0.055 (2)	0.0337 (18)	0.0026 (16)	−0.0032 (14)	0.0031 (16)
C10	0.0427 (19)	0.065 (3)	0.041 (2)	−0.0023 (18)	−0.0064 (16)	0.0121 (18)
C11	0.0354 (17)	0.063 (2)	0.0315 (19)	−0.0086 (16)	−0.0014 (14)	0.0019 (17)
C12	0.0399 (19)	0.063 (2)	0.0313 (19)	−0.0064 (17)	−0.0003 (14)	0.0004 (18)
C13	0.059 (2)	0.063 (3)	0.034 (2)	−0.003 (2)	0.0059 (17)	−0.0042 (18)
C14	0.068 (3)	0.068 (3)	0.051 (3)	−0.007 (2)	0.012 (2)	−0.011 (2)
C15	0.071 (3)	0.106 (4)	0.040 (3)	−0.005 (3)	−0.009 (2)	0.023 (3)
C16	0.060 (3)	0.108 (4)	0.030 (2)	−0.024 (3)	−0.0005 (17)	0.003 (2)
C17	0.050 (2)	0.061 (3)	0.042 (2)	−0.0113 (18)	0.0022 (16)	−0.0028 (17)

### Geometric parameters (Å, °)

**Table:**

S1—O1	1.418 (3)	C4—H4	0.9300
S1—O2	1.430 (3)	C5—C6	1.375 (6)
S1—N1	1.643 (3)	C6—C7	1.366 (6)
S1—C5	1.746 (4)	C6—H6	0.9300
S2—C10	1.722 (4)	C7—H7	0.9300
S2—C9	1.734 (4)	C8—C9	1.359 (5)
O3—C12	1.335 (5)	C8—C11	1.434 (5)
O3—C13	1.464 (4)	C9—C12	1.467 (5)
O4—C12	1.197 (5)	C10—C11	1.384 (6)
N1—C8	1.423 (5)	C11—C17	1.417 (5)
N1—H1N	0.95 (3)	C13—C14	1.496 (6)
N2—C15	1.340 (6)	C13—H13A	0.9700
N2—C10	1.342 (5)	C13—H13B	0.9700
C1—C2	1.532 (7)	C14—H14A	0.9600
C1—H1A	0.9600	C14—H14B	0.9600
C1—H1B	0.9600	C14—H14C	0.9600
C1—H1C	0.9600	C15—C16	1.355 (7)
C2—C7	1.390 (8)	C15—H15	0.9300
C2—C3	1.395 (8)	C16—C17	1.383 (6)
C3—C4	1.385 (6)	C16—H16	0.9300
C3—H3	0.9300	C17—H17	0.9300
C4—C5	1.390 (5)		
			
O1—S1—O2	119.11 (18)	C9—C8—N1	123.8 (3)
O1—S1—N1	107.37 (16)	C9—C8—C11	112.4 (3)
O2—S1—N1	105.00 (16)	N1—C8—C11	123.3 (3)
O1—S1—C5	109.43 (18)	C8—C9—C12	126.6 (3)
O2—S1—C5	107.97 (18)	C8—C9—S2	112.9 (3)
N1—S1—C5	107.34 (16)	C12—C9—S2	120.3 (3)
C10—S2—C9	90.63 (18)	N2—C10—C11	125.8 (4)
C12—O3—C13	116.1 (3)	N2—C10—S2	121.5 (4)
C8—N1—S1	122.0 (2)	C11—C10—S2	112.7 (3)
C8—N1—H1N	114 (3)	C10—C11—C17	118.2 (3)
S1—N1—H1N	109 (3)	C10—C11—C8	111.4 (3)
C15—N2—C10	114.2 (4)	C17—C11—C8	130.2 (4)
C2—C1—H1A	109.5	O4—C12—O3	124.0 (3)
C2—C1—H1B	109.5	O4—C12—C9	124.4 (4)
H1A—C1—H1B	109.5	O3—C12—C9	111.7 (3)
C2—C1—H1C	109.5	O3—C13—C14	107.3 (3)
H1A—C1—H1C	109.5	O3—C13—H13A	110.3
H1B—C1—H1C	109.5	C14—C13—H13A	110.3
C7—C2—C3	119.4 (5)	O3—C13—H13B	110.3
C7—C2—C1	121.5 (7)	C14—C13—H13B	110.3
C3—C2—C1	118.9 (6)	H13A—C13—H13B	108.5
C4—C3—C2	119.7 (5)	C13—C14—H14A	109.5
C4—C3—H3	120.1	C13—C14—H14B	109.5
C2—C3—H3	120.1	H14A—C14—H14B	109.5
C3—C4—C5	119.5 (5)	C13—C14—H14C	109.5
C3—C4—H4	120.3	H14A—C14—H14C	109.5
C5—C4—H4	120.3	H14B—C14—H14C	109.5
C6—C5—C4	120.8 (4)	N2—C15—C16	124.8 (4)
C6—C5—S1	119.7 (3)	N2—C15—H15	117.6
C4—C5—S1	119.5 (3)	C16—C15—H15	117.6
C7—C6—C5	119.7 (5)	C15—C16—C17	121.6 (4)
C7—C6—H6	120.1	C15—C16—H16	119.2
C5—C6—H6	120.1	C17—C16—H16	119.2
C6—C7—C2	120.9 (5)	C16—C17—C11	115.4 (4)
C6—C7—H7	119.6	C16—C17—H17	122.3
C2—C7—H7	119.6	C11—C17—H17	122.3
			
O1—S1—N1—C8	−38.8 (3)	C10—S2—C9—C12	−174.1 (3)
O2—S1—N1—C8	−166.5 (3)	C15—N2—C10—C11	3.0 (6)
C5—S1—N1—C8	78.8 (3)	C15—N2—C10—S2	−175.2 (3)
C7—C2—C3—C4	0.5 (7)	C9—S2—C10—N2	178.1 (4)
C1—C2—C3—C4	−174.8 (4)	C9—S2—C10—C11	−0.3 (3)
C2—C3—C4—C5	0.9 (7)	N2—C10—C11—C17	−2.6 (6)
C3—C4—C5—C6	−1.5 (6)	S2—C10—C11—C17	175.8 (3)
C3—C4—C5—S1	176.2 (3)	N2—C10—C11—C8	−178.3 (4)
O1—S1—C5—C6	−166.5 (3)	S2—C10—C11—C8	0.0 (4)
O2—S1—C5—C6	−35.5 (4)	C9—C8—C11—C10	0.4 (4)
N1—S1—C5—C6	77.3 (4)	N1—C8—C11—C10	172.4 (3)
O1—S1—C5—C4	15.8 (4)	C9—C8—C11—C17	−174.7 (3)
O2—S1—C5—C4	146.9 (3)	N1—C8—C11—C17	−2.7 (6)
N1—S1—C5—C4	−100.4 (3)	C13—O3—C12—O4	−0.1 (5)
C4—C5—C6—C7	0.7 (7)	C13—O3—C12—C9	178.8 (3)
S1—C5—C6—C7	−176.9 (3)	C8—C9—C12—O4	2.4 (6)
C5—C6—C7—C2	0.6 (7)	S2—C9—C12—O4	176.2 (3)
C3—C2—C7—C6	−1.2 (7)	C8—C9—C12—O3	−176.5 (3)
C1—C2—C7—C6	173.9 (4)	S2—C9—C12—O3	−2.7 (4)
S1—N1—C8—C9	−115.8 (4)	C12—O3—C13—C14	−178.0 (3)
S1—N1—C8—C11	73.0 (4)	C10—N2—C15—C16	−2.2 (7)
N1—C8—C9—C12	1.5 (6)	N2—C15—C16—C17	1.0 (7)
C11—C8—C9—C12	173.5 (3)	C15—C16—C17—C11	−0.4 (6)
N1—C8—C9—S2	−172.6 (3)	C10—C11—C17—C16	1.1 (5)
C11—C8—C9—S2	−0.6 (4)	C8—C11—C17—C16	175.9 (4)
C10—S2—C9—C8	0.5 (3)		

### Hydrogen-bond geometry (Å, °)

**Table:**

*D*—H···*A*	*D*—H	H···*A*	*D*···*A*	*D*—H···*A*
N1—H1N···O4	0.95 (3)	2.22 (3)	2.867 (4)	125 (3)
N1—H1N···O2^i^	0.95 (3)	2.21 (3)	3.033 (4)	145 (3)

Symmetry codes: (i) −*x*, −*y*+1, −*z*+1.
